# Influence of Nano, Micro, and Macro Topography of Dental Implant Surfaces on Human Gingival Fibroblasts

**DOI:** 10.3390/ijms22189871

**Published:** 2021-09-13

**Authors:** Morena Petrini, Tania Vanessa Pierfelice, Emira D’Amico, Natalia Di Pietro, Assunta Pandolfi, Camillo D’Arcangelo, Francesco De Angelis, Domitilla Mandatori, Valeria Schiavone, Adriano Piattelli, Giovanna Iezzi

**Affiliations:** 1 Department of Medical, Oral and Biotechnological Sciences, University G. d’Annunzio of Chieti-Pescara, Via dei Vestini 31, 66013 Chieti, Italy; morena.petrini@unich.it (M.P.); tania.pierfelice@unich.it (T.V.P.); emira.damico@unich.it (E.D.); assunta.pandolfi@unich.it (A.P.); camillo.darcangelo@unich.it (C.D.); francesco.deangelis@unich.it (F.D.A.); domitilla.mandatori@unich.it (D.M.); valeria.schiavone@unich.it (V.S.); adriano.piattelli@unich.it (A.P.); gio.iezzi@unich.it (G.I.); 2 Center for Advanced Studies and Technology-CAST (ex CeSI-MeT), University G. d’Annunzio of Chieti-Pescara, 66013 Chieti, Italy; 3 Research Center Fondazione Villa Serena per la Ricerca, 65013 Città Sant’Angelo, Italy; 4 Clinical Center, Casa di Cura Villa Serena del dott. L. Petruzzi, 65013 Città Sant’Angelo, Italy

**Keywords:** titanium, surface, roughness, macrogeometry, fibroblasts, dental implants

## Abstract

Current research on dental implants has mainly focused on the influence of surface roughness on the rate of osseointegration, while studies on the development of surfaces to also improve the interaction of peri-implant soft tissues are lacking. To this end, the first purpose of this study was to evaluate the response of human gingival fibroblasts (hGDFs) to titanium implant discs (Implacil De Bortoli, Brazil) having different micro and nano-topography: machined (Ti-M) versus sandblasted/double-etched (Ti-S). The secondary aim was to investigate the effect of the macrogeometry of the discs on cells: linear-like (Ti-L) versus wave-like (Ti-W) surfaces. The atomic force microscopy (AFM) and scanning electron microscopy (SEM) analysis showed that the Ti-S surfaces were characterized by a significantly higher micro and nano roughness and showed the 3D macrotopography of Ti-L and Ti-W surfaces. For in vitro analyses, the hGDFs were seeded into titanium discs and analyzed at 1, 3, and 5 days for adhesion and morphology (SEM) viability and proliferation (Cck-8 and MTT assays). The results showed that all tested surfaces were not cytotoxic for the hGDFs, rather the nano-micro and macro topography favored their proliferation in a time-dependent manner. Especially, at 3 and 5 days, the number of cells on Ti-L was higher than on other surfaces, including Ti-W surfaces. In conclusion, although further studies are needed, our in vitro data proved that the use of implant discs with Ti-S surfaces promotes the adhesion and proliferation of gingival fibroblasts, suggesting their use for in vivo applications.

## 1. Introduction

Dental implants have become a safe and reliable solution to replace missing teeth [1]. However, the long-term survival and success of implant therapy are influenced by various parameters [2,3]. A proper soft tissue seal between implants and gingiva represents one of these factors, operating as a protective barrier between the oral environment and the underlying peri-implant bone [4]. This connective tissue seal is particularly rich in fibroblasts that act against bacterial invasion, which may lead to unwanted clinical complications, such as inflammation, marginal bone resorption, and soft-tissue recession [5,6]. Therefore, in order to achieve ideal soft-tissue sealing, gingival fibroblasts (GFs) need to early adhere to the surfaces [7].

On the other hand, surfaces of dental implants are expected to demonstrate good soft-tissue biocompatibility. Titanium and titanium alloys are widely used biomaterials in the field of implantology because of their superior biocompatibility properties, and for their chemical and mechanical features [8]. In order to achieve good performance, some properties of the implant surfaces are considered critical issues for the host–implant integration. Therefore, a number of physicochemical modifications have been developed to improve surface cytocompatibility [9,10]. Several studies reported that hydrophilicity and surface roughness are critical parameters in the implant–tissue interaction and osseointegration. In particular, hydrophilic surfaces are more desirable than hydrophobic ones in view of their interactions with biological fluids, cells, and tissues [11,12,13]. The modification of the implant surface topography at nanoscale level has been largely investigated. Surface nanostructure can favorably influence cellular events at the titanium–bone interface, promoting the osseointegration [14,15,16]. Moreover, the bacterial interaction is influenced by the nano- and micro-topography of the fixture; the ideal material should promote the proliferation of mammalian cells, but without increasing the biofilm development [17,18].

While the research concentrated on the influence of the surface roughness on the rate of osseointegration, there are fewer studies focused on the development of surfaces that could also improve the peri-implant soft-tissue interaction [19].

A recent in vivo study on a novel dental implant, with a sandblasted and double etched surface and a macro-geometry characterized by the presence of decompression healing chambers, has shown promising results [20]. In particular, this novel macro-geometry seemed to enhance and accelerate the osseointegration.

Therefore, the objective of the present study was to analyze if and how the modifications in the surface characteristics of the titanium discs at the macro-, micro-, and nano-sized level could affect the biological activities of human gingival fibroblasts (hGDFs), considering that the macro topography is directly related to the implant geometry [21]. The primary outcome of the study was to analyze the effect of nano and microtopography of titanium discs on the hGDFs; the results of machined versus sandblasted and double etched discs were compared.

The second outcome was to evaluate the effect of the macro-geometry; two types of titanium discs, characterized by the same surface at the nano and micro-level, but with different macro-geometry, were compared. We examined the effects of these surface characteristics on the behavior of human gingival fibroblasts (hGDFs), concerning cell morphology, adhesion, viability, and proliferation.

## 2. Results

### 2.1. Surface Topography

**Figure 1:**
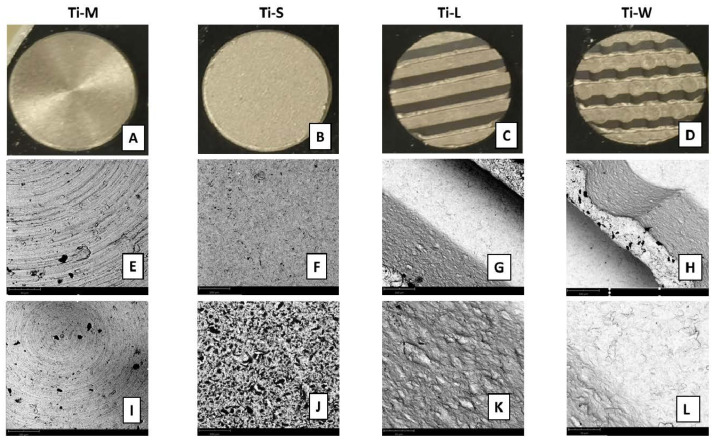
SEM observation of the four types of Ti discs used in the study (**A**–**D**). SEM morphologies of Ti-surfaces under different magnifications (**E**–**L**). Topographic macro characteristics were evidenced at low magnification of 290× (**E**–**H**). Notably, linear and wave macrogeometry of Ti-L and Ti-W substrates (**G**,**H**). Lower panels: the magnification at 1200× revealed the micro-topographic features of the discs (**I**–**L**) (scale bar: 200 μm at low magnification, 50 μm at high magnification).

**Figure 2:**
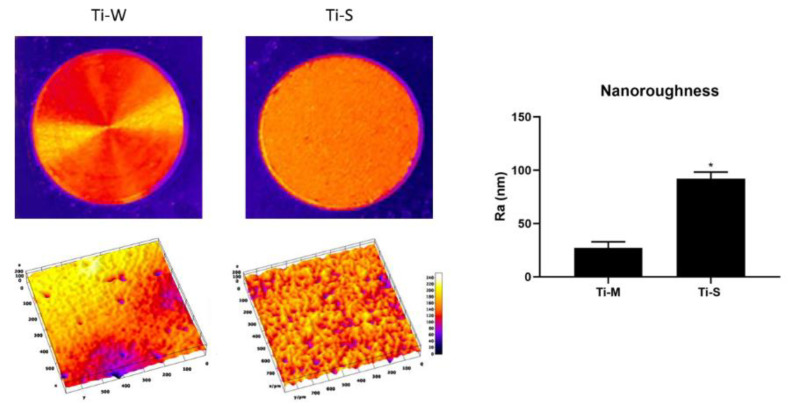
Macroscopical and microscopical 3D reconstruction and nano-analysis of Ti-M and Ti-S discs. The macroscopical and microscopical 3D reconstructions were obtained using ImageJ Software, starting from a photograph of the discs and SEM images at 295×, respectively. The nanoroughness measurements were obtained from AFM observations. The statistical analysis of the roughness average (Ra) expressed in nm evidenced that the roughness for the sandblasted/dual-etched surfaces (Ti-S) is higher with respect to the machined surface (Ti-M), (*denotes statistically significant differences between machined and sand-basted/dual-etched group, *p* < 0.001).

SEM images provided a macroscopic view of Ti-disc surfaces (Figure 1A–D). Images at 295× and 1200× magnifications showed that machined surfaces (Ti-M) were characterized by circular micro-threads and a mild roughness (Figure 1E,I and Figure 2). Sandblasted/dual-etched discs (Ti-S) exhibited visible topographic alterations owing to the treatment with titanium oxide particles and the double etching attack that significantly increased the superficial roughness, as shown in the 3D topography (Figure 2). The AFM analysis measured that sand-basted/dual-etched surfaces group (Ti-S) was characterized by a roughness average (Ra) of 92.030 ± 6.320 nm, whereas 27.285 ± 5.660 nm was the value for the machined surface (Ti-M) (Figure 2).

**Figure 3:**
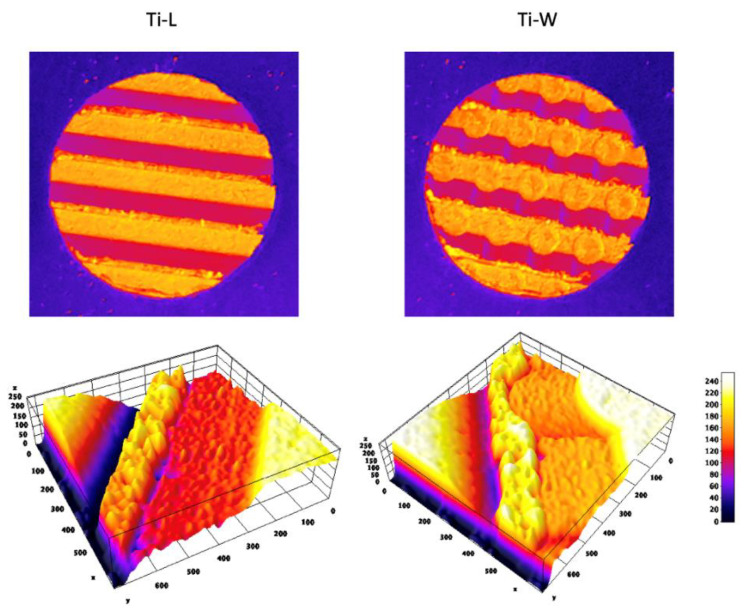
Macroscopical and microscopical 3D reconstruction Ti-M and Ti-S discs. The macroscopical and microscopical 3D reconstructions were obtained using ImageJ Software, starting from a photograph of the discs and SEM images at 295×, respectively.

The different macrogeometries that characterized Ti-L and Ti-W are shown in Figure 1G,H and Figure 3.

Lower magnification at 295× showed the 3D structures of sandblasted/dual-etched Ti-L and Ti-W surfaces with clearly identifiable macrogeometry (Figure 1G,H). Greater magnification 1200× of all sandblasted/dual-etched surfaces revealed the irregularities with pits and spikes of the area and the characteristic porous structures with the random arrangement of furrows of various sizes (Figure 1J–L). The filter applied on the macro-images of Ti-L and Ti-W and the respective 3D reconstruction of 295× SEM images permit to better observe the traditional linear threads of Ti-L and the presence of wave-like threads in the Ti-W (Figure 3).

**Figure 4:**
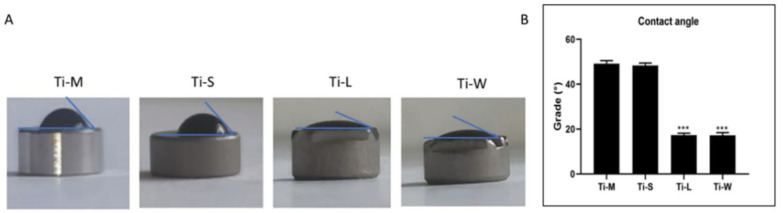
(**A**) Pictures of the WCA on the different surfaces, during the sessile drop method. (**B**) The average measured WCA (*** denotes statistically significant differences between machined and sand-basted/dual-etched group, *p* < 0.001).

The evaluation of the wetting properties (Figure 4A) confirmed that all surfaces could be considered as hydrophilic owing to their contact angle being smaller than 90°. Inside the sandblasted/dual-etched group, Ti-L and Ti-W had the lowest water contact angle WCA (17.3° and 17.2°, respectively), followed by Ti-S (48.4°), whereas the machined disc had the highest WCA (49.1°) (Figure 4B).

### 2.2. Cell Morphology and Adhesion

**Figure 5:**
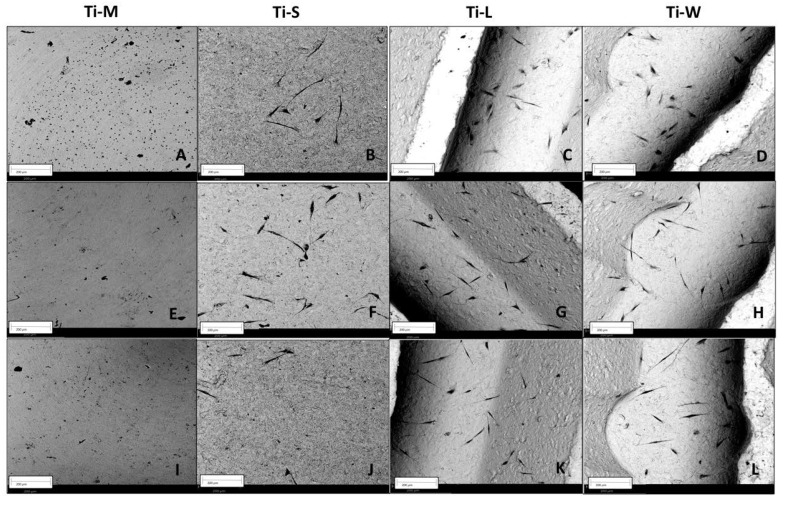
Morphology and adhesion of hGDFs on tested titanium surfaces at 290× magnification. Images show the round-shaped cells on Ti-M (**A**,**E**,**I**) and a visualization of filopodia spreading on sandblasted/dual-etched substrates: Ti-S (**B**,**F**,**J**), Ti-L (**C**,**G**,**K**), and Ti-W (**D**,**H**,**L**) at 1, 3, and 5 days, respectively. In addition to a typical morphology of fibroblasts, observed on TI-S discs, more cells remained adhered to the grooved structures covering the hollow portions of Ti-L and Ti-W surfaces. Especially at the fifth day, more adhered fibroblasts are observed on Ti-L (**K**) and Ti-W discs (**L**) with respect to the Ti-S surface (**J**). Scale bars: 200 μm. Images were taken using an accelerating voltage of 15 kV.

**Figure 6:**
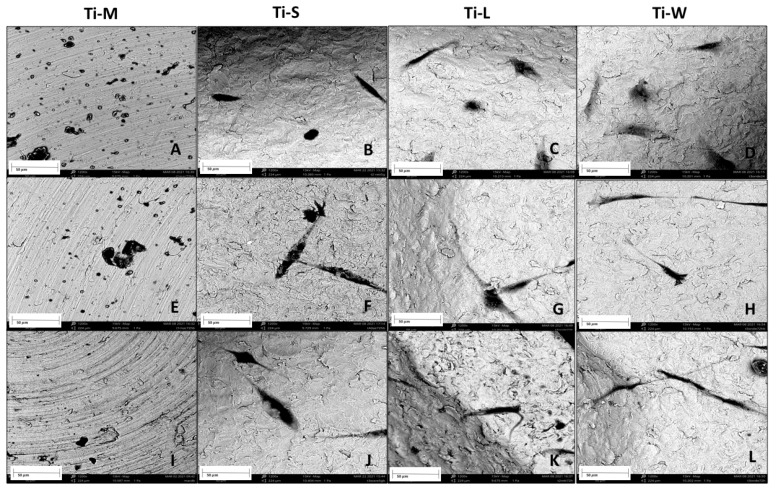
Morphology and adhesion of hGDFs on tested titanium surfaces at 1200× magnification. Images show the round-shaped cells on Ti-M (**A**,**E**,**I**) and a visualization of filopodia spreading on sandblasted/dual-etched substrates: Ti-S (**B**,**F**,**J**), Ti-L (**C**,**G**,**K**), and Ti-W (**D**,**H**,**L**) at 1, 3, and 5 days, respectively. Scale bars: 50 μm. Images were taken using an accelerating voltage of 15 kV.

The SEM analysis performed on fibroblast-seeded surfaces after 1, 3, and 5 days showed the effect of surface topography on morphology and adhesion of hGDFs (Figure 5 and Figure 6). Concerning the morphology, typically, polygonal spindle-shaped hGDFs attached and spread on all specimen surfaces with sandblasted and dual-etched treatment (Ti-S), while the cells seemed to be round-shaped on the machined surfaces (Figure 5A and Figure 6A). The apparent formation of filopodia and lamellipodia was observed only on sandblasted/dual-etched surfaces (Ti-S) at all the three timing-points (Figure 5 and Figure 6B–D,F–H,J–L). As observed by SEM images, cell adhesion on Ti-S was stronger than on Ti-M. Among the Ti-S surfaces, fibroblasts seemed to preferentially attach to grooved structures of Ti-L and Ti-W substrates. Some of them were observed in the hollow portion of macrogeometry-modified tested discs Ti-L and Ti-W (Figure 5 and Figure 6G,H). In addition, the macrogeometry seemed to favor the adhesion for a longer time. At 5 days, a higher number of cells remained attached to Ti-L and Ti-W substrates among to the Ti-S substrates group (Figure 5 and Figure 6J–L).

### 2.3. Cell Viability

**Figure 7:**
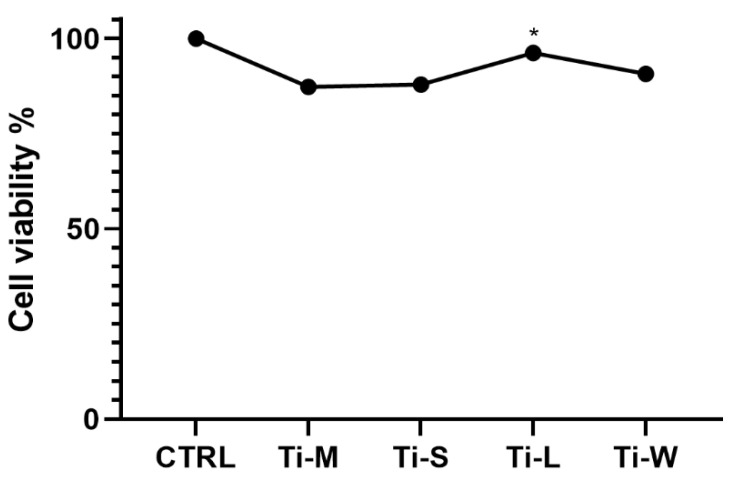
Effect of surface topography on hGDFs’ viability after 24 h of culture. More than 90% of cell viability was observed within all groups (* denotes the difference between the machined and sand-basted/dual-etched group, *p* < 0.05). There was no significant difference between the two surfaces with macrogeometry Ti-L and Ti-W.

Before setting up cell viability experiments, isolated hGDFs were characterized for their phenotype. As shown in Table 1, cells expressed the typical fibroblast cell markers CD105, CD73, and CD90, but not CD45 (leukocyte common antigen, LCA) or CD326 (epithelial cell adhesion molecule, EpCAM). The cell counting kit-8 (CCK-8) assay after 24 h of culture showed that all surfaces were fully biocompatible and exerted no negative effects on the viability of hGDFs (Figure 7). The significant difference among the surfaces was observed between the viability of fibroblasts seeded on Ti-L with respect to machined surfaces Ti-M, whereas no significant difference was observed between Ti-L and Ti-W surfaces.

### 2.4. Cell Proliferation

**Figure 8:**
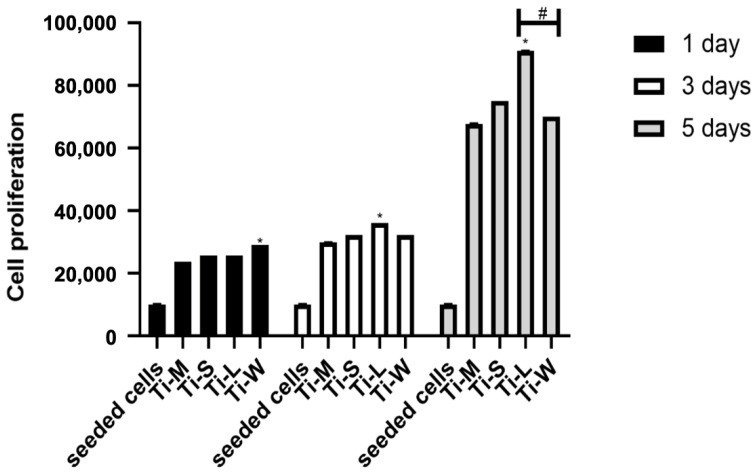
Comparison of fibroblast proliferation on Ti substrata after 1, 3, and 5 days of culture. Titanium disc surface (*n* = 4). The results are expressed in the form of number of cells. Seeded cells as the initial number of seeded human gingival fibroblasts 1 × 10^4^ cells/disc (* denotes the difference between the machined and sand-basted/dual-etched group, *p* < 0.05) (# denotes the difference between Ti-L and Ti-W, *p* < 0.05).

None of the Ti-surfaces modify the cell proliferation rate, which was time-dependent, as reported in the graph (Figure 8). However, the number of proliferative cells on Ti-S surfaces was higher compared with the machined surface Ti-M. Statistical differences were observed at all three timing-points: at day 1, the proliferative capabilities of cells seemed to be favored by the wave-like macrogeometry surface (Ti-W), whereas at days 3 and 5, the total cell metabolic activity on the linear-like macrogeometry surface (Ti-L) was significantly greater than on the other surfaces, including the wave-like macrogeometry surface (Ti-W).

## 3. Discussion

The long-term success of dental implants depends on the establishment and maintenance of both hard and soft peri-implant tissues [22,23]. Thus, numerous surface modifications of implant materials have been developed to ensure a good host-to-implant interface, and fixtures with multi-roughness surfaces have been proposed in order to take advantage of the specific features of the different parts of the fixtures [13,24,25]. Some authors proposed that an implant neck with smooth surfaces could be less plaque retentive compared with those with high roughness [26,27]. However, we largely studied the bacterial interaction with titanium surfaces and we showed that the biofilm accumulation is the result of many factors, at the nano-, micro-, and macro-scale, thus it is not possible to simplify this topic as merely a result of the material roughness [17,18,28,29,30]. The primary objective of this study was to evaluate the influence of nano- and micro-topography on hGDFs’ morphology, adhesion, and proliferation. Especially, the hGDFs’ response to sandblasted/dual-etched titanium surfaces (Ti-S) was compared with control (Ti-M). These cells have a critical role in the establishment and maintenance of an efficient soft-tissue seal around dental implants, producing several components of extracellular matrix and being involved in inflammatory response [31]. Ti-S surfaces were characterized by a significant higher nano- and micro-roughness compared with Ti-M, and the micro-topography also revealed the presence of irregularities with pits and spikes in the sandblasted discs. However, no significant differences were found for the wetting properties of the materials owing to the sandblasting treatment.

The SEM analysis performed on fibroblast-seeded samples after 1, 3, and 5 days showed how surfaces’ nano- and micro-topographies influence the adhesion, morphology, and proliferation of hGDFs. The average roughness (Ra) measurements demonstrated as sandblasted/dual etching treatment enhances the nano-roughness compared with machined surfaces, and this seems to improve the biocompatibility of the Ti-S surfaces group. Studies showed that nano-roughness is fundamental to increase the modulation of cell signaling and plays an important role in the regulating biomolecule adhesion behaviors [32,33,34]. In accordance with the higher level of nano-roughness, the increased porosity of sandblasted/dual-etched surfaces (Ti-S), observed at SEM without cells, and the micro-grooved surfaces promoted cell adhesion more than the smooth machined surfaces (Ti-M). These results are very encouraging because we have recently compared the bacterial and biofilm growth on machined and sandblasted surfaces, and no significant differences were found among the groups [30].

The differences we observed among the groups were also related to the morphology. The SEM observations typically showed polygonal, spindle-shaped cells, attached and spread on surfaces with sandblasted and dual etched treatment, with the formation of filopodia and lamellipodia, while cells appeared round-shaped on the machined surfaces. The morphology of cells observed as the black spots on the smooth surface of Ti-M was round-shaped at all time points. The cells cultured on Ti-M surfaces did not appear as flat as those on the other surfaces and diminished on days 3 and 5. This may suggest that the interaction between cells and the machined surfaces might be weaker than those on the surfaces with sandblasted and dual-etched treatment. After 24 h in culture, SEM revealed elongated, flat, large cells with a spindle-shape on Ti-S, Ti-L, and Ti-W surfaces, indicating good attachment. After 3 days in culture, spindle-shaped hGDFs were intimately attached and grown on all surfaces with sandblasted and dual-etched treatment. The cells started to stretch with the thin filopodia. After 5 days, gingival fibroblasts on Ti-S, Ti-L, and Ti-W discs were flat, elongated spindle-shaped cells possessing processes extending out from the ends of the cell body, which reflect a strong cell adhesion. The CCK-8 assay revealed that all samples did not have a cytotoxic effect on viability of hGDFs. Moreover, sandblasting/dual-etching treatments exert a more favorable biocompatibility than machined. These beneficial effects on the viability of fibroblasts were in line with the results of the MTT assay, which reported the cell proliferation activity. The number of proliferative cells on Ti-S surfaces group was higher compared with machined surface Ti-M for the entire trial, demonstrating greater cytocompatibility of sandblasted/dual-etched surfaces than the machined ones.

Studies showed that the implant macrogeometry represents one of the most important factors for successfully achieving primary stability [35,36]. For this reason, the secondary outcome was to evaluate the impact of macro-topography on Ti-S surfaces, so the hGDFs’ proliferation and viability were measured on these discs, characterized by an additional linear (Ti-L) and waved grooved macrogeometry (Ti-W). The cells on Ti-L and Ti-W surfaces were more numerous than on Ti-S surfaces, in accordance with the result of the proliferation study. This could suggest that macrogeometry might favor the cell adhesion. According to SEM images, the viability and proliferation graphs also indicated that these macro-topography modifications positively affected the behavior of cells more than the other experimented titanium surface Ti-M and Ti-S. In particular, the higher viability of fibroblasts detected for Ti-L was in accordance with a significantly enhanced cell metabolic activity of gingival cells seeded on the linear-like macrogeometry of Ti-L surface at days 3 and 5.

These results seem to be partly in contrast with previous in vivo studies, which showed that the dental implants with sandblasted and double-etched surface, marked by the presence of healing chambers, were characterized by a significant percentage of bone to implant contact (BIC%) and bone fraction occupancy inside the threads (BAFO%), with respect to those with traditional threads [21,37]. However, the in vivo results, as suggested by the same authors, are a consequence of less compressive trauma exerted by this novel macro-design on the bone, but in the in vitro conditions, this parameter is not present. Moreover, the presence of the free spaces between the bone and the implant with the healing chambers permits the accumulation of blood and the stabilization of fibroblasts, which is a prerogative for healing processes, as suggested by previous literature [38].

## 4. Material and Methods

### 4.1. Dental Implant

In the present study, four titanium disc surfaces (Implacil, DeBortoli, São Paulo, Brazil) were employed. All specimens were made of commercially pure titanium grade 4 (ASTM F67) and were equal in diameter and height, at 5 mm and 2 mm, respectively. These four different titanium discs were distinguished on the base of their nano and micro characteristics, into two groups: (*a*) machined discs group (Ti-M) had a machined surface and was cleaned with purified water, enzymatic detergent, acetone, acetyl acid (double acid attack), and alcohol; (*b*) sandblasted followed by dual acid etched discs group (Ti-S) had a surface treatment of sand blasting that was made with a mix of titanium oxide powder, followed by cleaning with purified water, enzymatic detergent, acetone, acetylic acid (double acid attack), and alcohol. The Ti-S group was further distinguished on the base of the macro-topography as (*i*) linear-like macrogeometry (Ti-L): traditional linear macro-design of dental implants (macrogeometry of Due Cone De Bortoli implants); or (*ii*) wave-like macrogeometry (Ti-W): new macro-geometry implant with the presence of decompression chambers (macrogeometry of De Bortoli Maestro implants).

### 4.2. Surface Characterization

#### 4.2.1. AFM Analysis for Nanoscale Characterization

The average nano-roughness (Ra) of machined and sandblasted/dual-etched discs was measured under atomic force microscopy (AFM, Bruker). The ScanAsyst technique was used for the atomic force microscopy observations with a scan size of 10 µm × 10 µm and a RTESPA-300 probe. The roughness average (Ra), which is the arithmetic mean of the absolute values of the height of the surface profile, was considered for the statistical analysis.

#### 4.2.2. SEM Analysis for Microscale Characterization

The SEM observation was performed both on untreated surfaces and on those with cultivated cells.

Surface topography was studied only on untreated discs (Phenom-World BV, Eindhoven, The Netherlands). ImageJ Software 1.52 q (National Institute of Health, Bethesda, MD, USA) with the SurfCharJ and 3D reconstruction plugins were used to characterize the disc’s surface from macropictures and SEM images at 295× magnification. Before the observation of the discs covered with cells, all the tested substrates (*n* = 4) were fixed with 2.5% glutaraldehyde solution and rinsed in PBS and dehydrated at increasing alcohol concentrations (35%, 50%, 70%, 95%, and 100%) for 30 min each, followed by 100% hexamethyldisilazane (HMDS, Sigma-Aldrich, St. Louis, MO, USA), overnight. Discs were then mounted onto aluminum stubs and gold-coated in an DSR1Desk Sputter Coater/Carbon Evaporator (Nanostructured Coatings Co., Tehran, Iran), before imaging by means of SEM. Images were taken using an accelerating voltage of 15 kV with the backscattered electronic signal detector (BSE), BSD full, to obtain images of cells of a different color (black) than the titanium surface (gray).

#### 4.2.3. Wettability Analysis for Macroscale Characterization

The surface wettability of Ti-discs in each surface was also examined using the sessile drop technique, as previously described [17,18]. Briefly, 1 uL of saline solution was pipetted on each disc and, immediately, a Nikon D90 DSLR camera (Nikon Corporation, Tokyo, Japan) with an 18–105 mm lens was used to photograph the samples. The water contact angle was then measured using ImageJ 1.52 q for Mac OS X (USA).

### 4.3. Biological Analysis

#### 4.3.1. Cell Culture

Seven human gingival biopsies were obtained from partial gingivectomy procedures of patients treated in the dental clinic of the University G. D’Annunzio Chieti-Pescara (CE, N° 1968-24 July 2020). For hGDFs’ isolation, gingival biopsies underwent a double enzymatic digestion for 1 h at 37 °C using a solution containing collagenase type 1A and dispase (both from Sigma-Aldrich).

Subsequently, residual gingival samples were placed in a petri dish with D-MEM low glucose added with 10% of FBS (cat.41A0045K, Life Technologies, Budapest, Hungary), 1% P/S (cat. P4333, Sigma-Aldrich, St. Louis, MO, USA), and 100 mM L-Glu (cat. G7513, Sigma-Aldrich, St. Louis, MO, USA) to obtain a final spontaneous migration of hGDFs. Isolated cells were grown in a controlled atmosphere (5% CO_2_ and 37 °C) up to the confluence and used for all experiments between the 3° and 6° passage.

The phenotypic characterization of hGDFs was performed by cytometric analysis through the expression of CD105 (FITC-conjugated antibody; Becton Dickinsons BD Bioscience, San Diego, CA, USA cat.326-040), CD73 (PE-conjugated antibody; Becton Dickinsons BD Bioscienc, San Diego, CA, USA cat.550257), CD90 (FITC-conjugated antibody; Becton Dickinsons BD Bioscience, San Diego, CA, USA cat.555595), CD326 (PerCP-Cy5.5-conjugated antibody; Becton Dickinsons BD Bioscience, San Diego, CA, USA, cat.347199), and CD45 (FITC-conjugated antibody; Becton Dickinsons BD Bioscience, San Diego, CA, USA, cat.196-040). FACSVerse (BD Bioscences, San Diego, CA, USA), FACSDiva v 6.1.3, IDEAS software (BD Biosciences, San Diego, CA, USA), and FlowJo 8.3.3 software (Tree Star Inc., Ashland, OR, USA) were used for the analysis.

#### 4.3.2. Evaluation of Morphology and Adhesion

The effects of surface topography on cell adhesion and shape were analyzed by SEM, and then hGDFs were cultivated on the Ti discs for 1, 3, and 5 days. The cells were seeded at a density of 1 × 10^4^ cells/disc. The cultured cells were incubated for timing-points at 37 °C in 5% CO_2_. Loosely adherent cells were removed from the experiment wells by washing twice with a 0.1 M PBS (pH 7.4), and the remaining bound cells were fixed as previously explained. Subsequently, the samples were dried and then gold-coated using Emitech K550 (Emitech Ltd., Ashford, UK) sputter-coater before imaging by means of SEM (Philips XL20; Philips Inc., Eindhoven, The Netherlands).

#### 4.3.3. Cell Viability Assay

The cell viability of hGDF cells cultured on the discs’ surfaces was assessed by Cell Counting Kit–8 (Sigma Aldrich, St. Louis, Missouri, USA). Here, 10^4^ cells/well were seeded on the top of the disc into a 96-well culture plate with DMEM low glucose and 10% FBS. After 24 h of incubation, 10 μL of the CCK-8 solution was added to each well and the plate was further incubated for 2 h. The absorbance at 450 nm was measured using a microplate reader (Synergy H1 Hybrid BioTek Instruments, Winooski, VT, USA). The results were then expressed in the form of percentage compared with cells (100%) seeded onto the well without any titanium disc (CTRL). The assay was assessed in five replicates and three independent analyses.

#### 4.3.4. Cell Proliferation Assay

hGDF cells were seeded on top of each specimen at a density of 1 × 10^4^ cells/well. The hGDFs were incubated in direct contact with the disc for 1, 3, and 5 days. At the end of each incubation period, a solution of 0.5 mg/mL MTT (Sigma Aldrich, St. Louis, MO, USA) was added to each well and then the cells were incubated for 4 h at 37 °C and 5% CO_2_. A solubilization solution was added into each well to dissolve the insoluble formazan. The spectrophotometrical absorbance of the samples was measured at 650 nm using a microplate reader (Synergy H1 Hybrid BioTek Instruments, Winooski, VT, USA). Five replicates and three independent analyses were assessed for MTT assay.

### 4.4. Statistical Analysis

All experiments were performed in 3–5 replicates and repeated three times. For comparing between two groups, T test was used. Specifically, for the primary outcome, machined and sandblasted/dual-etched discs were compared. For the secondary outcome, linear-like microgeometry (Ti-L) and wave-like microgeometry (Ti-W) were compared. Statistical analysis was performed using GraphPad Prism 8. A value of *p* < 0.05 was considered statistically significant. A value of *p* < 0.001 was considered highly statistically significant.

## 5. Conclusions

In this study, we found a significant increase in cellular activity in all discs treated with sandblasting and double-etching treatment compared with machined. This cellular activity seemed to be correlated with the increase in the nano-roughness and micro-geometry noticed on sandblasted and dual-etched surfaces. The macrogeometry seemed further to favor the fibroblast activity, mainly the adhesion, morphology, and proliferation. On the contrary, we found no significant differences among the two macro-geometries; both surfaces enhanced the biological response of gingival cells, except for the proliferation, which was significantly higher for linear grooved macrogeometry (Ti-L) surfaces at 5 days after cell cultivation.

## Figures and Tables

**Table 1:** Flow cytometry analysis of the hGDFs’ phenotype.

Antigenes	Expression Levels
CD 73	+
CD 90	+
CD 105	+
CD 45	−
CD 326	−

− negative expression; + positive expression.
